# Does cigarette reduction while using nicotine replacement therapy prior to a quit attempt predict abstinence following quit date?

**DOI:** 10.1111/add.13330

**Published:** 2016-03-16

**Authors:** Nicola Lindson‐Hawley, Bethany Shinkins, Robert West, Susan Michie, Paul Aveyard

**Affiliations:** ^1^ Nuffield Department of Primary Care Health SciencesRadcliffe Observatory QuarterUniversity of OxfordOxfordUK; ^2^ UK Centre for Tobacco and Alcohol StudiesUniversity of NottinghamNottinghamUK; ^3^ Health Behaviour Research CentreDepartment of Epidemiology and Public HealthUniversity College LondonLondonUK; ^4^ Centre for Behaviour ChangeUniversity College LondonLondonUK

**Keywords:** NRT, observational, RCT, Reduction, smoking cessation, tobacco addiction

## Abstract

**Background and Aims:**

Previous studies have reported that people who use a smoking cessation medication while smoking and reduce cigarette consumption spontaneously are three times more likely to stop smoking after a quit date. The aim was to replicate this and assess whether it arises because of willed effortful reduction rather than unwilled reduced drive to smoke caused by medication.

**Design:**

Secondary analysis of a trial where participants were randomised to smoke as normal or reduce by 75% over 2 weeks prior to quit date, using nicotine replacement therapy (NRT) in both arms.

**Setting:**

Thirty‐one UK primary care practices.

**Participants:**

A total of 517 adult smokers seeking quitting support in the carbon monoxide (CO) analyses and 421 in the cigarettes/day analyses.

**Measurements:**

Russell Standard abstinence was recorded 4 weeks after quit date. The randomized groups were combined and the association between reduction and abstinence examined. The second analysis assessed whether this association differed by whether smokers were, or were not, instructed to reduce.

**Findings:**

In all participants, there was no evidence that reducing cigarettes/day or CO by at least half compared with not reducing predicted abstinence at 4 weeks [risk ratio (RR) = 0.88; 95% confidence interval (CI) = 0.68–1.14 and RR = 1.20; 95% CI = 1.00–1.44, respectively]. However, in smokers instructed to reduce, CO reduction was associated with 4‐week abstinence (RR = 1.52; 95% CI = 1.16–2.00), but not among people advised not to reduce (RR = 0.91; 95% CI = 0.67–1.24).

**Conclusions:**

Smoking reduction prior to a target quit date while on a smoking cessation medication may only predict subsequent abstinence when smokers are consciously attempting to reduce.

## Introduction

An important problem for the science and practice of smoking cessation is that there appears to be little basis for matching treatments to particular patients. One promising approach that might allow personalization could be for smokers to try medication prior to attempting to stop smoking and to choose medication based on their response. Response to medication may be indicated by spontaneous reduction in smoking, either by reduced daily consumption of cigarettes or other markers of smoke intake, such as concentration of exhaled carbon monoxide or cotinine concentration.

Several studies have examined whether or not spontaneous smoking reduction while using smoking cessation medication is associated with a higher likelihood of quitting smoking after a quit date. In four of these trials, participants used nicotine replacement therapy (NRT) for 2 weeks prior to quitting 1, 2, 3, 4. Participants who reduced consumption by more than the median (56%) while smoking and using NRT were found in one study to be more than three times more likely to stop smoking than people reducing less than the median 3. Similarly, in people using varenicline for 4weeks prior to quit day, those who reduced consumption (indicated by cotinine concentration) by more than 50% were approximately three times more likely to achieve abstinence than those not reducing by this amount 5. However, in three cases of the aforementioned studies 1, 2, 5 observations were *post hoc*, which makes interpretation difficult. Evidence in a similar field suggests that initial findings of observational associations tend to overestimate the strength of association 6.

The first aim of this study is to conduct a planned replication study to examine whether smoking reduction while using cessation medication prior to quit date predicts abstinence. The second aim is to examine two plausible mechanisms that might explain this association. The explanation favoured by the investigators in the aforementioned studies 1, 2, 3, 5 is that smoking reduction is unwilled and occurs because medication met the need to smoke, and hence will support smokers adequately after they try to stop smoking. However, there is a second possible explanation. The investigators in these previous studies advised participants to smoke freely. It could be that participants on cessation medication chose to try to reduce their smoking. According to this explanation, participants who were successful at controlling their smoking and manifesting a reduction would become successful when they try to quit because the same forces are at play during successful reduction as successful cessation. In support of this, a systematic review suggests that successful willed reduction is associated causally with subsequent cessation 7. We exploited the design of a recent trial to examine these competing explanations.

## Method

### Data source and permissions

Data were obtained through a non‐inferiority randomized controlled trial (RCT) of abrupt cessation versus smoking reduction prior to cessation conducted in England from 2009 to 2012 (trial registration number: ISRCTN22526020) 8. The main finding of the trial was that abrupt quitting resulted in superior 4‐week quit rates to quitting smoking following smoking reduction [relative risk (RR) = 0.80; 95% confidence interval (CI) = 0.67, 0.95]. The trial was authorized by the National Research Ethics Committee, the Medicines and Healthcare products Regulatory Agency and local NHS Research and Development offices. Separate permissions were not required for this analysis.

### Design

The Rapid Reduction Trial (RRT) was a randomized, non‐inferiority trial of 697 participants with two trial arms. In the reduction trial arm participants were asked to reduce their smoking to 50% of their baseline rate in the first week after baseline, and to 75% of their baseline smoking rate in the second week following baseline, before quitting completely. Participants were advised to choose and follow one of three structured reduction plans. More details of these methods are available in the trial protocol 8. During the 2‐week pre‐quit phase, reducers used nicotine patches (21 mg, reducing to 14 mg in the event of intolerance to the higher dose) and an acute form of NRT (i.e. gum, lozenge etc.) to compensate for the missed cigarettes. In the abrupt trial arm participants were also asked to set a quit day for 2 weeks after baseline, but were advised to smoke as they usually would and not reduce for the 2 weeks before quitting completely. In this group participants used nicotine patches only (21 or 14 mg) prior to the quit date. We did not give participants acute NRT, as this group were not intentionally trying to reduce smoking.

In both groups participants were provided with weekly behavioural support from 2 weeks prior to quit day to 4 weeks after quit day and then again at 8 weeks. In the 2 weeks prior to quit day, the reduction group discussed reduction strategies and progress made, and we addressed barriers to reduction. In the abrupt arm participants were prepared for quitting by asking them to think about scenarios that they may find difficult after the quit day and thinking of potential ways to deal with these if they should arise. After quit day, the treatment in both arms was identical. Participants used combination NRT (i.e. 21‐mg nicotine patches and an acute form of NRT) with weekly behavioural support focused on dealing with cravings and withdrawal and preventing relapse.

We exploited this design in an observational analysis to examine our competing hypotheses. First, we examined overall whether degree of reduction while smoking and using NRT was associated with future cessation. Secondly, we used the difference in instructions by trial arm to examine the degree to which intentional reduction was associated with cessation success. In the abrupt arm, the instruction was ‘try to smoke as normal’ but, as anticipated, not everyone could smoke the same number of cigarettes while using NRT. People who reduced despite advice to smoke the same number were probably doing so because they felt impelled to do so by the medication effect. In the other (reduction) arm, the instruction was to try to reduce smoking, and therefore the degree of reduction in this arm probably represents the ability to achieve intentional control over smoking. This difference in instruction differentiates between competing hypotheses in the way that the *ad‐libitum* smoking instruction in previous studies has not.

### Participants

Twenty‐three nurses recruited people who smoked in 31 primary care practices in the West Midlands of England. Randomization was stratified by nurse and each nurse randomized between 6 and 120 participants. General practitioners (GPs) wrote to their patients who smoked asking them if they would like to quit smoking and, if so, to contact the trial team. Trial clinics took place in participants’ GP practices. Participants were eligible if they met the following criteria: smoking at least 15 cigarettes per day (CPD); willing to stop smoking completely in 2 weeks; not currently undergoing any other treatment to stop smoking; and no medical reasons that would mean concurrent smoking and use of NRT was inadvisable. Almost all people with medical, psychiatric and comorbid substance use problems were enrolled.

### Variables

The following variables collected during RRT were relevant to the reported analysis.

#### Reduction in smoking

We measured the number of cigarettes smoked and the concentration of exhaled carbon monoxide (CO) at baseline (visit 1) and in the following 2 weeks, prior to quit day (at visit 2; a week after baseline; and visit 3, 2 weeks after baseline, the day before quit day). For each participant we calculated the percentage change in baseline CPD and CO between visits 1 and 3. We also dichotomized these variables because reduction by at least 50% has been used previously as, or has been found to be, an indicator of response to medications in research studies 3, 5.

#### Smoking cessation

Abstinence data were collected at 4‐week and 6‐month follow‐ups (measured from quit day). In both cases abstinence was defined using the Russell Standard (RS) approach—intention‐to‐treat, assuming those lost to follow‐up resumed smoking, allowing a grace period of 2 weeks after quit day, with no more than five cigarettes smoked thereafter, and validated by an exhaled CO reading of <10 parts per million (p.p.m.) 9.

#### Potential confounders

The following variables were potential confounders, as they may be associated with the likelihood of smoking cessation: gender; age (in years); ethnicity (dichotomized as white ethnicity or other); post‐school qualification (dichotomized as having a post‐school qualification or not); employment (dichotomized as in paid employment or not); age started smoking (in years); nicotine dependence at baseline [measured using the Fagerström Test for Cigarette Dependence (FTCD)] 10, 11; baseline saliva cotinine (measured in ng/ml); number of previous quit attempts; length of longest abstinence achieved in a previous quit attempt (dichotomized as less than a month or longer); living with smoker or not; confidence in quitting at baseline (measured on the following response scale: low, not very high, quite high, very high, extremely high); trial arm (reduction versus abrupt); and pre‐randomization trial arm preference (reduction arm, abrupt arm, no preference).

### Analysis

Some people did not complete the daily diary, which recorded cigarette consumption, and hence data on reduction in cigarettes were missing. Some did not attend the visit 2 weeks after baseline (the day before quit day), and hence data on CO reduction were missing. We examined whether there were systematic differences between the people who did not supply data on reduction and those who did by comparing medians and proportions using χ^2^ tests for categorical baseline variables and Mann–Whitney *U*‐tests for ordinal or continuous variables.

The strength of association between change in smoking measured using CO and CPD and abstinence at 4 weeks and 6 months was explored. We present relative risks with 95% CIs due to the high incidence of abstinence (> 10%), using a modified Poisson generalized estimating equation using the glm command in STATA 12. Models were run with CO and CPD as continuous variables and dichotomized variables. These analyses were repeated adjusting for potential confounders. The impact of the nurse (the stratification factor in the trial) was explored in the main trial analyses, and no evidence of clustering was found; therefore it was not adjusted for in this analysis.

We examined whether the strength of association differed by trial arm by including appropriate multiplicative interaction terms for the continuous reduction variables. As multiple tests were carried out, a *P*‐value of 0.01 or less indicated a significant interaction. Regardless of the significance of this term, we presented the strength of the association by trial arm. We could not include the interaction term for the dichotomous reduction measures due to high collinearity, and so instead we ran separate models for each trial arm to produce relative risk estimates.

## Results

### Baseline characteristics of those in the analyses

A total of 697 participants were enrolled into the trial and therefore provided baseline data. There were some differences between the whole population recruited and the sample who supplied data on reduction and were included in these analyses. Participants supplying data for the current analyses [421 (60.4%) for the CPD analyses and 517 (74.2%) participants for the CO analyses] were slightly more likely to be allocated to the abrupt trial arm (CPD 56.3%; CO 56.3 versus 50.9% of the total participants randomized into the trial) (Table 1). Also, as expected, people not attending clinic visits prior to quit day or not completing diaries were less likely to achieve abstinence; therefore the abstinence rates in the samples used in the current analyses are higher than those observed in the total trial sample.

**Table 1:** Participant characteristics.

*Baseline participant characteristic*	*All randomized participants*			*CPD analysis*			*CO analysis*		
	*Total (n = 697)* a	*Abrupt arm (n = 355)*	*Reduction arm (n = 342)*	*Total (n = 421)* a	*Reduced <50% (n = 208)* a	*Reduced ≥50% (n = 213)* a	*Total (n = 517)* a	*Reduced <50% (n = 336)* a	*Reduced ≥50% (n = 181)* a
Abrupt trial arm, *n/N* (%)	355/697 (50.9)	355/355 (100.0)	0/342 (0.0%)	237/421 (56.3)	185/208 (88.9)	52/213 (24.4)	291/517 (56.3)	242/336 (72.0)	49/181 (27.1)
Age, median (IQR)	49.0 (17.0)	49.0 (17.0)	49.0 (17.3)	50.0 (18.0)	50.0 (17.0)	50.0 (19.0)	50 (18)	50.0 (17.0)	48.0 (17.5)
Male gender, *n/N* (%)	350/697 (50.2)	175/355 (49.3)	175/342 (51.2)	234/421 (55.6)	114/208 (54.8)	120/213 (56.3)	276/517 (53.4)	177/336 (52.7)	99/181 (54.7)
White ethnicity, *n/N* (%)	648/692 (93.6)	329/351 (93.7)	319/341 (93.5)	398/419 (95.0)	198/206 (96.1)	200/213 (93.9)	480/513 (93.6)	313/333 (94.0)	167/180 (92.8)
Post‐secondary school (15/16 years) educational qualification, *n/N* (%)	345/678 (50.9)	185/348 (53.2)	160/330 (48.5)	215/410 (52.4)	110/202 (54.5)	105/208 (50.5)	259/505 (51.3)	164/332 (49.4)	95/173 (54.9)
In paid employment, *n/N* (%)	382/691 (55.3)	192/351 (54.7)	190/340 (55.9)	240/419 (57.3)	112/206 (54.4)	128/213 (60.1)	298/513 (58.1)	187/333 (56.2)	111/180 (61.7)
Age started smoking (years), median (IQR)	16.0 (4.0)	16.0 (4.0)	16.0 (3.0)	16.0 (3.0)	16.0 (3)	16.0 (3)	16.0 (3.0)	16.0 (3.0)	16.0 (3.0)
Lives with smoker, *n/N* (%)	266/688 (38.7)	150/353 (42.5)	116/335 (34.6)	160/418 (38.3)	118/206 (57.3)	140/212 (66.0)	196/514 (38.1)	139/334 (41.6)	57/180 (31.7)
Number of previous quit attempts, median (IQR)	2.0 (2.0)	2.0 (3.0)	2.0 (2.0)	2.0 (2.0)	2.0 (2.0)	2.0 (2.0)	2.0 (2.0)	2.0 (2.0)	2.0 (2.0)
Longest previous quit attempt < a month, *n/N* (%)	275/660 (41.7)	144/337 (42.7)	131/323 (40.6)	167/398 (42.0)	82/197 (41.6)	85/201 (42.3)	204/490 (58.4)	135/318 (42.5)	69/172 (40.1)
Longest previous quit attempt ≥ a month, *n/N* (%)	385/660 (58.3)	193/337 (57.3)	192/323 (59.4)	231/398 (58.0)	115/197 (58.4)	116/201 (57.7)	286/490 (58.4)	183/318 (57.5)	103/172 (59.9)
Salivary cotinine concentration (ng/ml), median (IQR)	358.5 (212.7)	349.5 (197.7)	365.3 (234.5)	367.5 (201.0)	379.2 (183.9)	365.2 (218.5)	360.3 (200.8)	367.2 (200.3)	352.4 (208.3)
Cigarettes per day (CPD), median (IQR)	20.0 (10.0)	20.0 (9.0)	20.0 (10.0)	20.0 (10.0)	20.0 (8.0)	20.0 (10.0)	20.0 (10.0)	20.0 (8.0)	20.0 (7.0)
Fagerström Test for Nicotine Dependence (FTND) score, median (IQR)^b^	6.0 (3.0)	6.0 (3.0)	6.0 (3.0)	5.0 (3.0)	6.0 (3)	5.0 (3)	6.0 (3.0)	6.0 (3)	5.0 (3)
Preference for abrupt treatment arm, *n/N* (%)	224/697 (32.1)	117/355 (33.0)	107/342 (31.3)	126/420 (30.0)	60/207 (29.0)	66/213 (31.0)	166/516 (32.2)	110/335 (32.8)	56/181 (30.9)
Preference for gradual treatment arm, *n/N* (%)	355/697(50.9)	176/355 (49.6)	179/342 (52.3)	214/420 (51.0)	106/207 (51.2)	108/213 (50.7)	255/516 (49.4)	162/335 (48.4)	93/181 (51.4)
No trial arm preference, *n/N* (%)	118/697 (16.9)	62/355 (17.5)	56/342 (16.4)	80/420 (19.0)	41/207 (19.8)	39/213 (18.3)	95/516 (18.4)	63/335 (18.8)	32/181 (17.7)
Confidence in quitting, median (IQR)^c^	4.0 (1.0)	4.0 (1.0)	4.0 (1.0)	4.0 (1.0)	4.0 (1.0)	4.0 (1.0)	4 (1.0)	4.0 (1.0)	4.0 (1.0)

a Numbers of participants used to calculate statistics for each variable vary due to missing data.

b Range from 0 to 10, where 10 = highest level of dependence.

c Measured on a scale from 1 to 6, where 1 = very low and 6 = extremely high. IQR = interquartile range; CO = carbon monoxide; CPD =cigarettes per day; FTND = Fagerstrom Test for Nicotine Dependence.

We compared those who supplied data for these analyses to those who did not do so. People supplying data were significantly more likely to be male for both the CPD and CO analyses (CPD 55.6 versus 42.0% male; CO 53.4 versus 41.1%) and less heavily dependent upon cigarettes in the CO analysis only (median FTND 5 versus median FTND 6).

In the case of both the CPD and CO analyses, just more than half the participants were male (234 of 421, 55.6% and 276 of 517, 53.4%, respectively), the average age was 50 years in both groups, participants had an average baseline salivary cotinine concentration of 368 and 360 ng/ml and an FTND of 5 and 6, respectively (Table 1). At 4‐week follow‐up 240 of 421 (57.0%) participants were abstinent in the CPD analysis and 289 of 517 (55.9%) in the CO analysis, and at 6‐month follow‐up 100 of 421 (23.8%) in the CPD analysis and 118 of 517 (22.8%) in the CO analysis (Table 2).

**Table 2:** Reduction and abstinence outcomes in the whole sample.

*Participant smoking outcome*	*All (n = 697)* a	*CPD analysis (n = 421)* a	*CO analysis (n = 517)* a
Average percentage CPD reduction over pre‐quit period, median (IQR)	49.0 (48.9)	50.0 (50.3)	49.0 (48.9)
Reduced CPD by ≥50% during pre‐quit period, *n/N* (%)	213/421 (30.6)	213/421 (50.6)	200/400 (50.0)
Average percentage CO reduction over pre‐quit period, median (IQR)	35.7 (50.2)	35.7 (50.2)	34.5 (51.5)
Reduced exhaled CO by ≥50% during pre‐quit period, *n/N* (%)	181/517 (26.0)	142/400 (35.5)	181/517 (35.0)
Abstinent at 4 weeks post‐quit, *n/N* (%)	308/697 (44.2)	240/421 (57.0)	289/517 (55.9)
Abstinent at 6 months post‐quit, *n/N* (%)	131/697 (18.8)	100/421 (23.8)	118/517 (22.8)

CPD = cigarettes per day; CO = carbon monoxide; IQR = interquartile range.

a Numbers of participants used to calculate statistics for each variable vary due to missing data.

### Association between pre‐cessation reduction and abstinence in the whole sample

Participants randomized to the reduction arm of the trial reduced cigarette consumption and exhaled CO levels during the 2 weeks by a mean of 69 and 47%, respectively. Participants randomized to the abrupt arm also reduced their consumption by a mean of 29 and 21%. Participants in the reduction arm were more likely to drop out prior to quit day, probably because they were failing to achieve reduction.

There was no association between reduced cigarette consumption and smoking abstinence at 4 weeks or 6 months (Table 3). At 4 weeks, 124 of 213 (58.2%) participants who reduced their CPD by at least 50% and 116 of 208 (55.8%) of participants who reduced their CPD by less than 50% were abstinent, and at 6 months 50 of 213 (23.5%) participants who reduced their CPD by at least 50% and 50 of 208 (24.0%) of participants who reduced their CPD by less than 50% were abstinent.

**Table 3:** The association between reduction over a 2‐week pre‐quit period and smoking abstinence at 4 weeks and 6 months post‐quit.

	*Relative risk (RR) of abstinence at 4 week follow‐up RR (95% CI)*		*RR of abstinence at 6 month follow‐up RR (95% CI)*	
	*Unadjusted analyses*	*Adjusted analyses* a	*Unadjusted analyses*	*Adjusted analyses* a
Reduction in CPD^b^	1.02 (0.99–1.05)	1.01 (0.97–1.05)	1.02 (0.96–1.07)	1.00 (0.93–1.08)
At least 50% reduction in CPD	1.04 (0.88–1.23)	0.88 (0.68–1.14)	0.98 (0.69–1.38)	0.76 (0.45–1.29)
Reduction in CO^c^	1.02 (0.99–1.04)	1.04 (1.01–1.06)	1.01 (0.98– 1.05)	1.01 (0.96–1.06)
At least 50% reduction in CO	1.17 (1.01–1.37)	1.20 (1.00–1.44)	1.23 (0.89–1.69)	1.39 (0.97–2.00)

a All adjusted for gender; age; ethnicity; post‐school qualification; employment; age started smoking; Fagerstrom Test for Nicotine Dependence score; baseline saliva cotinine (measured in ng/ml); number of previous quit attempts; length of longest quit attempt; living with smoker; confidence in quitting at baseline; trial arm; pre‐randomization trial arm preference.

b Relative risk presented for a 10% cigarette per day reduction.

c Relative risk presented for a 10% reduction in CO.

RR = relative risk; CI = confidence intervals; CPD = cigarettes per day; CO = carbon monoxide.

There was a modest significant association between the degree of reduction in exhaled CO levels and smoking abstinence at 4‐week follow‐up. For every 10% reduction in baseline CO participants achieved, the likelihood of cessation at 4 weeks increased by 4% after adjustment. Dichotomizing change in CO to more than 50% reduction or less showed that at 4 weeks, 112 of 181 (61.9%) participants who reduced their CO by at least 50% and 177 of 336 (52.7%) participants who reduced their CO by less than 50% were abstinent; the difference was not significant before or after adjustment. There were no associations with 6‐month abstinence. At 6 months, 47 of 181 (26.0%) participants who reduced their CO by at least 50% and 71 of 336 (21.1%) participants who reduced their CO by less than 50% were abstinent.

### Do the different instructions modify the association between reduction and subsequent cessation?

There was no statistically significant evidence that the instructions given to participants in each trial arm modified the strength of association between degree of reduction and subsequent cessation (Table 4). None the less, it is noteworthy that the risk ratio was larger in the reduction arm than the abrupt arm in all eight comparisons. In the abrupt arm, which represented normal smoking cessation practice, there was no evidence of an association between reduction and subsequent cessation. This was apparent only in the reduction arm on some measures: the arm in which participants were aiming to reduce.

**Table 4:** The adjusted association between smoking reduction and abstinence at 4 weeks and 6 months post‐quit, split by trial arm (abrupt versus reduction).

	*4‐week follow‐up*			*6‐month follow‐up*		
	*Risk of abstinence in abrupt arm RR (95% CI)*	*Risk of abstinence in reduction arm RR (95% CI)*	*Significance of interaction effect trial arm × reduction*	*Risk of abstinence in abrupt arm RR (95% CI)*	*Risk of abstinence in reduction arm RR (95% CI)*	*Significance of interaction effect trial arm × reduction*
Reduction in CPD^a^	0.99 (0.95–1.04)	1.06 (0.98–1.15)	*P* = 0.17	0.98 (0.84–1.14)	1.00 (0.92–1.10)	*P* = 0.75
At least 50% reduction in CPD^b^	0.77 (0.55–1.06)	1.10 (0.66–1.85)	NA	0.60 (0.28–1.26)	1.09 (0.41–2.92)	NA
Reduction in CO^c^	1.02 (0.99–1.05)	1.06 (1.02–0.11)	*P* = 0.09	0.99 (0.94–1.05)	1.05 (0.93–1.18)	*P* = 0.41
At least 50% reduction in CO^b^	0.91 (0.67–1.24)	1.52 (1.16–2.00)	NA	0.86 (0.46–1.60)	2.17 (1.13–4.16)	NA

a Relative risk presented for a 10% cigarette per day reduction.

Interaction term could not be included due to collinearity and therefore separate models were run for each trial arm.

Relative risk presented for a 10% reduction in CO.

RR = relative risk; CI = confidence intervals; CPD = cigarettes per day; CO = carbon monoxide; NA = not available.

## Discussion

In this planned replication study there was little evidence overall that smoking reduction while using nicotine replacement therapy and smoking predicted subsequent abstinence. We hypothesized that the association between reduction and quitting reported previously 1, 2, 3, 5 could have arisen, because people felt that they ought to reduce smoking intentionally while using cessation medication. We did not find strong evidence that the strength of association between reduction and cessation varied by trial arm. Nevertheless, an association between reduction and subsequent cessation was manifest in the condition where participants deliberately reduced their smoking.

An important strength of this study is that it is a planned replication of previous *post‐hoc* findings. As in the other studies, this is an observational analysis comparing naturally occurring groups (reducers with non‐reducers across trial arms), albeit within the setting of an RCT, and therefore subject to potential for confounding. However, we adjusted for many potential confounders and adjustment did not change the findings greatly, so it is unlikely that confounding obscured the association between reduction and subsequent cessation. It may have been that the instruction to participants in the abrupt arm to smoke as normal may have deterred medication use, but there was no evidence of this. Patch use was very similar in both trial arms, with between 80 and 90% of participants using their patches daily. Additionally, our advice to smoke as usual in the abrupt cessation arm may have prevented reduction being manifest. Despite this advice, however, 81 and 69% of the participants in this arm reduced CPD and CO, respectively, suggesting that our advice did not prevent reduction. Finally, we have inferred that people in the reduction arm achieved their reduction partly through efforts to do so, and not through the additional short‐acting NRT available in this arm. We believe this is reasonable, given that the typical dose was two pieces of gum/lozenge per day. We have also inferred that people who reduced in the arm who were advised that it would be helpful not to reduce were doing so because they felt a reduced drive to smoke engendered by the medication, rather than because they were wilfully doing so. These inferences seem logical, but are not supported by direct observations of whether people were trying to reduce or not.

The trial followed participants to 6 months after quitting, which allowed us to ascertain that, although there was one significant association between reduction and cessation at 4 weeks, this was not observed at 6 months. Therefore, this may have been a chance finding. The estimates were precise enough to exclude effects of the size reported previously, which suggested a threefold difference in the likelihood of achieving abstinence 3, 5.

The cut‐off used to identify responders to medication is a 50% reduction, based on a median split used previously in one study 3 and rounded to 50% in another 5. We also examined change in CPD and CO as continuous variables, which generally showed no relationship with cessation. Our findings stand in stark contrast to these previous findings, in showing no overall evidence of a relationship between reduction and subsequent cessation while using smoking cessation medication. Unlike previous studies, the design of our trial allowed us to split the sample into those who were reducing because they felt impelled to do so by medication and those who were trying to reduce. In the main, previous studies have instructed participants to smoke freely. By so doing, we found that the association seemed to be apparent only among those who were trying to reduce. This raises the possibility that the findings in these previous studies may have been due to people trying to reduce. It seems likely that people who can control their smoking more successfully when reducing can enact these same strategies to abstain completely after quit day.

The best evidence that smoking reduction on medication indicates a higher likelihood of achieving abstinence would come from an adaptive clinical trial. In such a trial, smokers who want to quit smoking would try several cessation medications to identify the one to which they respond by reducing when allowed to smoke freely. This would be compared with a non‐adaptive treatment approach, where cessation medication is picked at random. There are two such adaptive trials [4,13]; the first randomized people who did not reduce consumption by 50% while using a nicotine patch to quit on a nicotine patch, add bupropion to the patch or switch to varenicline 4. The quit rates on both varenicline and bupropion plus NRT were higher, but this was significant only in the bupropion condition. However, these results are clouded by the fact that evidence suggests there may be a benefit of bupropion plus NRT over NRT alone (although this is uncertain) 14, and that varenicline appears to be more effective than NRT 15, suggesting that the results may be due to more efficacious medication *per se* rather than tailoring to the response to NRT itself. In the second trial, participants were asked to use varenicline for 3 weeks prior to quitting 13. Those in whom smoking was not suppressed were randomized to either continue on the standard dose or have the dose of varenicline increased. There was no evidence of benefit from dose escalation. Our results may help to explain this. The initial observations, that smoking reduction while smoking freely and using pharmacotherapy was associated with subsequent cessation, may have been published because the association was unexpectedly large, but also plausible and potentially useful. Our planned replication suggests that this marker may be a weak predictor of response to medication, and it may not be as useful as first thought, in line with the trial findings.

It is important to note what we are not saying with these data. We observed that people advised to reduce and who did so appeared more likely to go on to quit smoking, while people who were instructed to maintain but in fact reduced were not more likely to quit smoking. It could be surmised that advising people to reduce while using pre‐quit NRT is helpful and advising people not to reduce is unhelpful, but this would be incorrect. The trial from which these data are obtained showed that people randomized to the arm in which people were advised to try to smoke as normal were 25% more likely to stop smoking in the short and long term. Secondly, a systematic review 7 suggests that smoking reduction may itself make cessation more likely, and our data are not contrary to that. Trials of smoking reduction interventions enrol people who are making effortful attempts to reduce smoking. If anything, our data reinforce that causal association by showing an apparent dose–response relation between effortful reduction and subsequent cessation. They do, however, imply that unwilled reduction that appears to occur in response to medication may be predictive of cessation only weakly or not at all, and therefore attempts to tailor medication based on people's responses while smoking may be futile. This is unfortunate, particularly because attempts to rescue failing quit attempts after the quit date appear to be unsuccessful 4, 16. The strategy of selecting an optimum treatment based on pre‐cessation response is attractive but appears, on the basis of these data, to be unhelpful.

In conclusion, reduced consumption while using nicotine replacement and smoking may be a less strong indicator of response to medication than thought previously. Indeed, it may instead simply reflect people's efforts to reduce smoking. The search for a reliable indicator of which cessation medication may prove effective for particular individuals should continue.

### Clinical trial registration

Registered on the International Standard Randomised Controlled Trial Number Register before the start of participant enrolment (ISRCTN22526020). Available online at: http://controlled‐trials.com/ISRCTN22526020

### Declaration of interests

N.L.H., B.S. and S.M. have none to disclose; R.W. undertakes research and consultancy for companies that manufacture smoking cessation medications, including NRT. He is honorary co‐director of the UK's national Centre for Smoking Cessation and Training and a trustee of the charity, QUIT; P.A. reports personal fees and payment to the University of Oxford from Pfizer.
